# From low remission to hope: the efficacy of targeted therapies in NUP98-R positive pediatric acute myeloid leukemia

**DOI:** 10.1007/s12519-025-00875-w

**Published:** 2025-02-27

**Authors:** Run-Ji Xiong, Hong-Xia Tang, Tian-Tian Yin, Hui-Yi Pan, Run-Ming Jin

**Affiliations:** Department of Pediatrics, Union Hospital, Tongji Medical College, Huazhong University of Science and Technology, No.1277, Jiefang Avenue, Wuhan, 430022 China

**Keywords:** Acute myeloid leukemia, Pediatric, Targeted therapies, Treatment

## Abstract

**Background:**

Treating pediatric acute myeloid leukemia (AML) with NUP98 rearrangement (NUP98-R) is challenging. Standard chemotherapy results in low remission rates. This study aimed to evaluate different induction regimens and explore alternative therapies to improve outcomes.

**Methods:**

This retrospective study included 111 pediatric patients with AML treated at our institution from March 2012 to March 2023. Patients were classified into two groups: NUP98-R-positive (*n* = 10) and NUP98-R-negative (*n* = 101). We compared their clinical characteristics, treatment responses, and prognoses. Additionally, we presented three cases of NUP98-R-positive patients to elaborate on the role of targeted therapies during induction in treatment outcomes and prognosis.

**Results:**

Patients with NUP98-R fusion genes had a complete remission (CR) rate of 20% after the first induction, which was significantly lower than the 64.3% reported in those without NUP98-R fusion genes (*P* < 0.05). The 3-year event-free survival (EFS) rate was also lower, with only 30% for NUP98-R patients and 55.3% for non-NUP98-R patients (*P* < 0.05). The prognosis of NUP98-R patients improved with targeted therapies during induction. For example, Patient 1 achieved CR with FLT3 and BCL-2 inhibitors plus conventional chemotherapy. Patient 2, who was treated with a CDK6 inhibitor, a BCL-2 inhibitor, azacitidine, and an FLT3 inhibitor, also achieved CR and underwent successful stem cell transplantation. Conversely, Patient 3, who received only standard chemotherapy, did not achieve remission and died from a severe infection.

**Conclusions:**

This study demonstrated that using targeted drugs for the induction in NUP98-R pediatric AML improved treatment outcomes. BCL-2, FLT3, and CDK6 inhibitors available at our institution are promising options for this phase of treatment.

**Graphical abstract:**

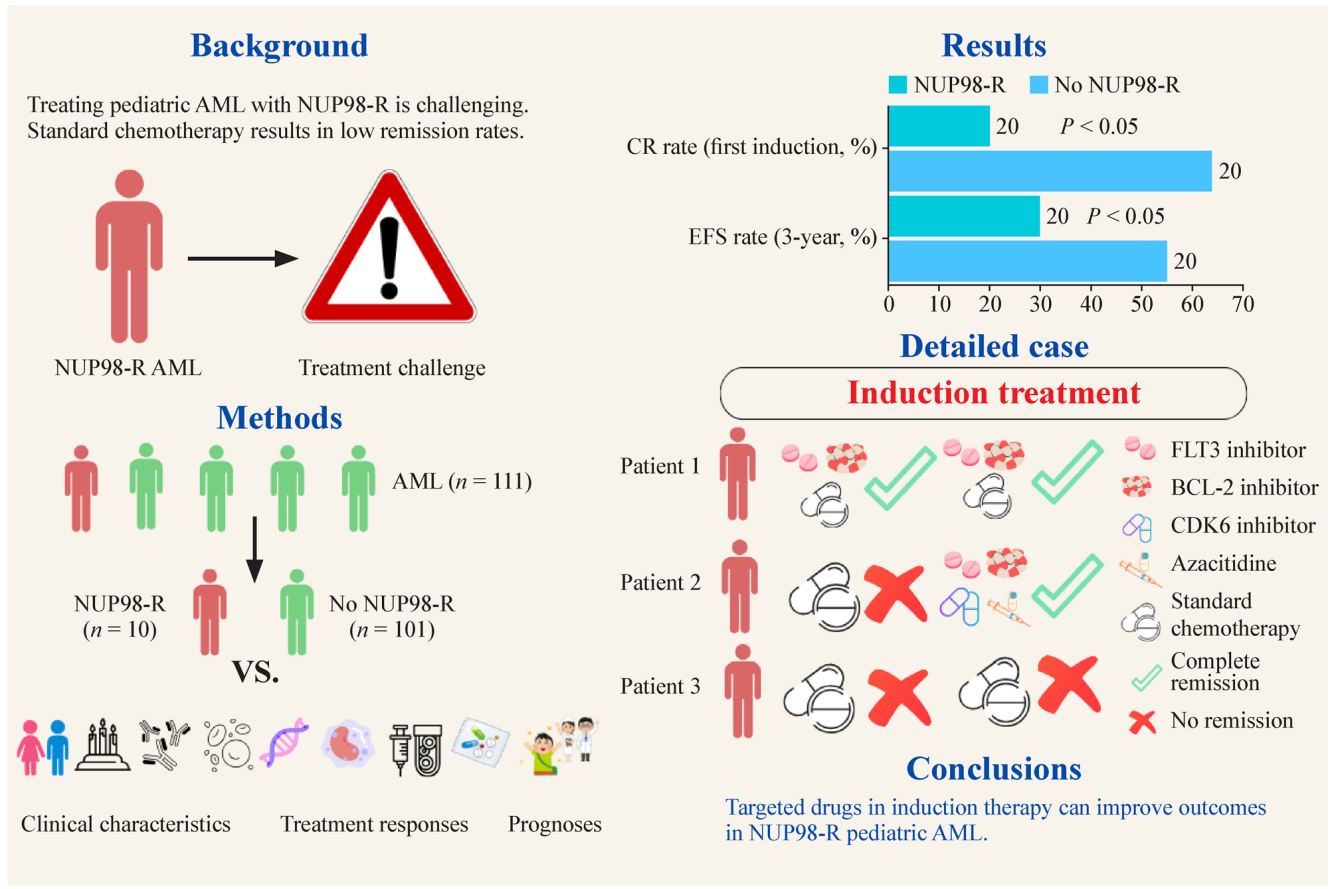

## Introduction

NUP98 gene rearrangements occur in 3.8%–7% of pediatric acute myeloid leukemia (AML) patients, with over 30 fusion partners identified; NUP98-NSD1 is the most common type [1, 2]. Patients with NUP98 rearrangement (NUP98-R) have a complete remission (CR) rate of about 50% after standard induction therapy, with a 5-year overall survival (OS) rate of 30%–35% [1, 3]. Notably, patients with both NUP98-NSD1 and FLT3-ITD mutations have a poorer prognosis, with a CR rate of only 30% and a relapse rate of 80%–90% [4]. The NUP98 fusion protein interacts with various proteins. This interaction regulates gene expression via a phase-separated mechanism, contributing to leukemogenesis [5]. The NUP98-NSD1 fusion promotes leukemogenesis by interacting with MLL1, resulting in H3K36 methylation and activation of *HOXA* genes, suggesting a link to hypermethylation in NUP98-R AML [6]. Animal models coexpressing NUP98-NSD1 and FLT3-ITD have demonstrated elevated *HOXA/B* gene expression and sensitivity to FLT3 inhibitors [7, 8]. Recent case reports have highlighted favorable outcomes with targeted therapies, such as FLT3 inhibitors and demethylating agents [9, 10]. Despite these promising outcomes, effective treatment options are still under exploration, and no standardized protocol has been established. In light of these challenges, this study compiles clinical data from 111 children with AML treated at our institution between 2012 and 2023 and presents three cases that illustrate diverse treatment strategies for potential consideration.

## Methods

### Participants and study design

This retrospective study enrolled children with AML treated at our institution between March 2012 and March 2023. AML diagnoses were based on morphology, flow cytometry, immunohistochemistry, and genetic testing, following the latest WHO pediatric classification system [11]. The inclusion criteria were as follows: (1) aged < 18 years at diagnosis and (2) AML diagnosis confirmed at our institution. The exclusion criteria were as follows: (1) incomplete clinical data and (2) patients who were untreated after diagnosis. Follow-up was conducted via outpatient visits or phone consultations until January 2024.

A total of 111 patients were included and divided into NUP98-R-positive (*n* = 10) and NUP98-R-negative (*n* = 101) groups. Clinical characteristics, treatment responses, and prognoses were compared between the groups. The study was approved by the Ethics Committee of the Union Hospital of Tongji Medical College, Huazhong University of Science and Technology (No. 2024–0753). Written consent for the publication of the details of three cases was obtained from the patients' parents.

### Genetic analysis

Bone marrow (BM) samples from pediatric patients were collected via PAXgene Blood RNA Tubes. Genomic DNA was extracted with the QIAamp DNA Blood Mini Kit. For targeted analysis of structural variants (SVs) and single-nucleotide variants (SNVs), a customized panel of biotinylated oligoprobes (Roche NimbleGen) was designed to capture likely gene breakpoints and hotspot mutations in hematological malignancies identified in prior leukemia studies.

### Definitions

BM evaluations were performed on day 28 of each chemotherapy cycle. CR was defined as the presence of less than 5% blasts in the BM and the regeneration of normal hematopoietic cells. OS was defined as the time from diagnosis to death or the last follow-up. Events included relapse, death, and loss to follow-up due to disease progression, abandonment of treatment, or unstable vital signs. Patients who completed most of the chemotherapy and achieved remission but were subsequently lost to follow-up were not considered events. Event-free survival (EFS) was defined as the time from treatment initiation to the first occurrence of any event or the last follow-up.

### Statistical analysis

Baseline patient characteristics are presented as frequencies and percentages for categorical data and as medians with interquartile ranges for continuous variables. Categorical variables were analyzed with chi-square tests, whereas non-parametric tests were used for continuous variables. OS and EFS were estimated via the Kaplan‒Meier method. Continuous variables included age, BM and white blood cell (WBC) count, whereas categorical variables included sex, age group, French–American–British classification, chromosomal aberrations, molecular genetics, stem cell transplantation status, CR at the end of course 1, and minimal residual disease (MRD) positivity at the end of course 1. *P* values were calculated via two-tailed tests, with *P* < 0.05 indicating statistical significance. Analyses were conducted via SPSS (IBM, version 24.0), Prism (version 9), and R (version 4.3.3).

### Clinical characteristics

**Fig. 1:**
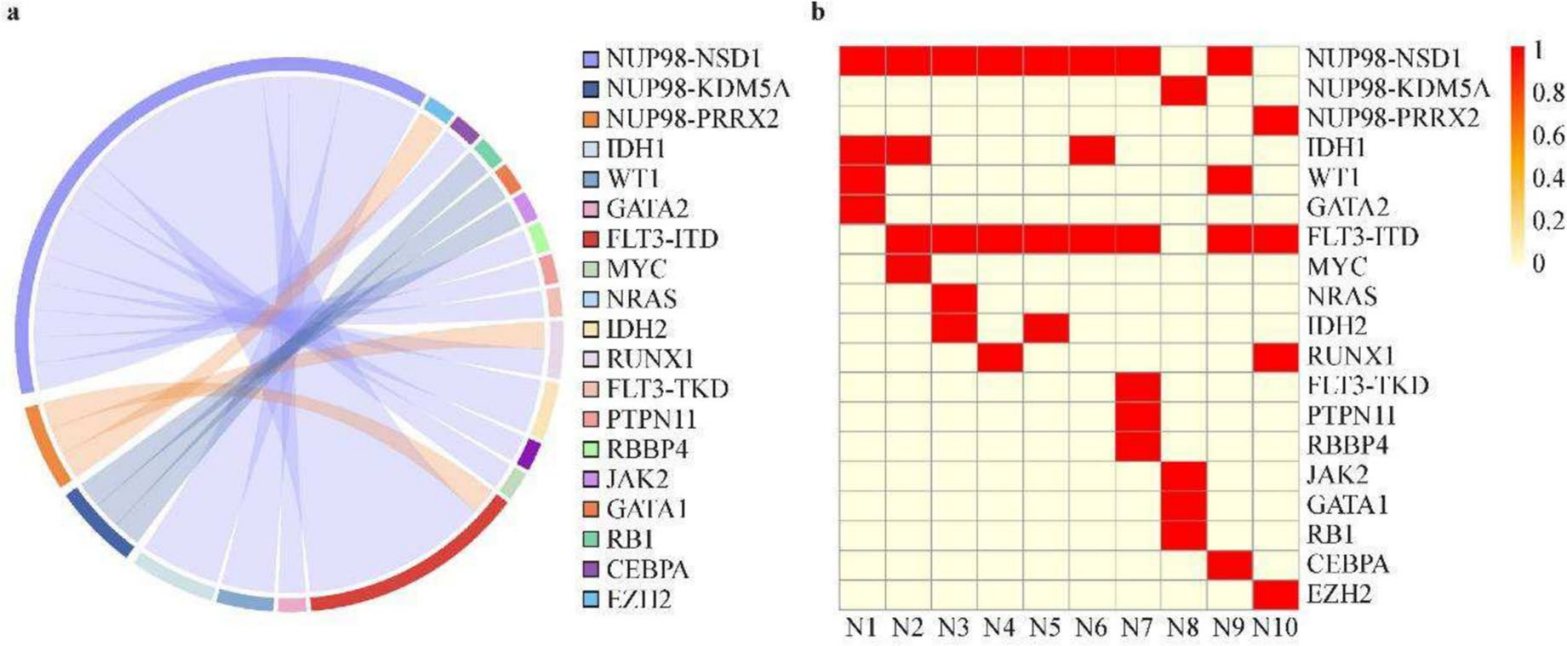
Displaying co-occurring mutations and fusion partner genes of NUP98-R pediatric AML patients. **a** Chord diagram depicting commonly cooccurring mutations in NUP98-translocated pediatric AML patients. **b** Heatmap depicting the distribution of fusion and mutant genes associated with each patient who tested positive for NUP98-R

Among the 111 patients, 10 had NUP98 translocations, whereas the remaining 101 formed the reference cohort. Patients in the NUP98-R group had significantly greater WBC counts (134.09 vs. 16.02 × 10^9^/L, *P* < 0.05) and BM blast percentages (78.25% vs. 57.5%, *P* < 0.05) than did those in the reference cohort (Table 1). The mutation burden was also greater in the NUP98-R group, with FLT3-ITD mutations found in 80% of patients compared with 14.8% in the reference group (*P* < 0.05). Similarly, IDH1/2 mutations were more common in the NUP98-R subgroup (50% vs. 11.8%, *P* < 0.05). Notably, 60% of NUP98-R patients (6/10) had a normal karyotype at diagnosis, which differed from that in the reference cohort. The most common NUP98 fusion subtype was NUP98-NSD1, which was present in 8 of 10 cases (80%). Other fusion partners included KDM5A and PRRX2 (Table 2, Fig. 1). The NUP98-R group also had a male predominance, with eight males (80%) and two females (20%) (Table 1).

**Table 1:** Clinical characteristics of the 111 patients

Characteristics	No NUP98 fusion (*N* = 101)	NUP98 fusion (*N* = 10)	*P*
Median age	7	5.2	0.086
(range)	(5, 10)	(3.14, 8)	
Sex, *N* (%)			0.38
Male	61 (60)	8 (80)	
Female	40 (40)	2 (20)	
Age category, *N* (%)			
< 3 y	11 (10.9)	2 (20)	0.735
3–10 y	67 (66.3)	7 (70)	1
> 10 y	23 (22.8)	1 (10)	0.594
FAB, *N* (%)			
M2	55 (54.4)	1 (10)	< 0.05
M4	16 (15.8)	3 (30)	0.488
M5	16 (15.8)	3 (30)	0.488
M7	6 (5.9)	0 (0)	1
WBC (× 10^9^/L), median	16.02	134.09	< 0.05
(range)	(5.84, 56.02)	(72.64, 267.54)	
BM, %	57.5	78.25	< 0.05
(Range)	(40, 80)	(70.75, 89)	
Chromosomal aberrations, *N* (%)			
Normal	25 (24.8)	6 (60)	< 0.05
t (6;9)	1 (1)	0 (0)	1
t (8;21)	31 (30.7)	0 (0)	0.09
inv (16)	9 (8.9)	0 (0)	0.706
Monosomy 5/del5q	0 (0)	0 (0)	1
Del7q	1 (1)	0 (0)	1
Monosomy 7	2 (2)	0 (0)	1
Trisomy 8	4 (4)	0 (0)	1
Chromosomal 13			
Abnormal chr13	2 (2)	1 (10)	0.639
chr13 deletion (del13q)	1 (1)	1 (10)	0.425
Molecular genetics, *N* (%)			
FLT3-ITD	15 (14.8)	8 (80)	< 0.05
WT1	15 (14.8)	2 (20)	1
CEPBA	11 (10.9)	1 (10)	1
KIT	24 (23.8)	0 (0)	0.181
CBL	4 (4)	0 (0)	1
GATA2	19 (18.8)	1 (10)	0.795
NRAS	22 (21.8)	1 (10)	0.64
IDH1/2	12 (11.8)	5 (50)	< 0.05
SCT yes, *N* (%)	40 (40)	4 (40)	1
CR end course 1, *N* (%)	65 (64.3)	2 (20)	< 0.05
MRD^+^ end course 1, *N* (%)	52 (51.4)	8 (80)	0.164

*FAB* French-American-British classification, *Abnormal chr13* Including del13q, monosomy 13, and translocations involving chromosome 13, *MRD*^+^ measurable residual disease positivity (measured by flow cytometry), *SCT* stem cell transplantation, *CR* complete remission (measured by morphology), *WBC* white blood cell count

**Table 2:** Clinical characteristics and treatment outcomes of ten patients with NUP98-R positive

ID	Sex/Age (y)	Partner gene	FLT3-ITD	Treatment	CR	Outcomes
N1	M/3.4	NSD1	N	DAH → IAH → DCAG + Ven → HSCT	Y	Survival
N2	M/11	NSD1	Y	DAH + Sor + Ven → IAH + Sor + Ven → HSCT	Y	Death
N3	M/2	NSD1	Y	DAH + Sor	NA	Give up
N4	M/8	NSD1	Y	DAH → IAH	Y	Death
N5	M/8	NSD1	Y	DAH → Ven + Aza	N	Give up
N6	M/5	NSD1	Y	DAH → Gil + Pal + Aza + Ven → HSCT	Y	Survival
N7	M/6.1	NSD1	Y	DAH → IAH → CLAG	N	Death
N8	M/2.8	KDM5A	N	DAH	N	Give up
N9	F/5.4	NSD1	Y	DAH → IAH → FLAG	N	Death
N10	F/3.2	PRRX2	Y	DAE → DAC + AA → HSCT	Y	Survival

*CR* complete remission (prior to HSCT or last follow-up), *F* female, *M* male, *NA* data not available, *N* No, *Y* Yes, *DAH* Daunorubicin + Cytarabine + Homoharringtonine, *IAH* idarubicin + Cytarabine Homoharringtonine, *DCAG* Decitabine + Cytarabine + Aclarubicin + Granulocyte Colony-Stimulating Factor, *Sor* Sorafenib, *Ven* Venetoclax, *Aza* Azacitidine, *Gil* Gilteritinib, *Pal* Palbociclib, *CLAG* Cladribine + Cytarabine + Granulocyte Colony-Stimulating Factor, *FLAG* Fludarabine + Cytarabine + Granulocyte Colony-Stimulating Factor, *DAE* Daunorubicin + Cytarabine + Etoposide, *DAC* + *AA* Daunorubicin + Aclarubicin + Cytarabine, *HSCT* hematopoietic stem cell transplantation;

### Clinical outcomes and prognosis

**Fig. 2:**
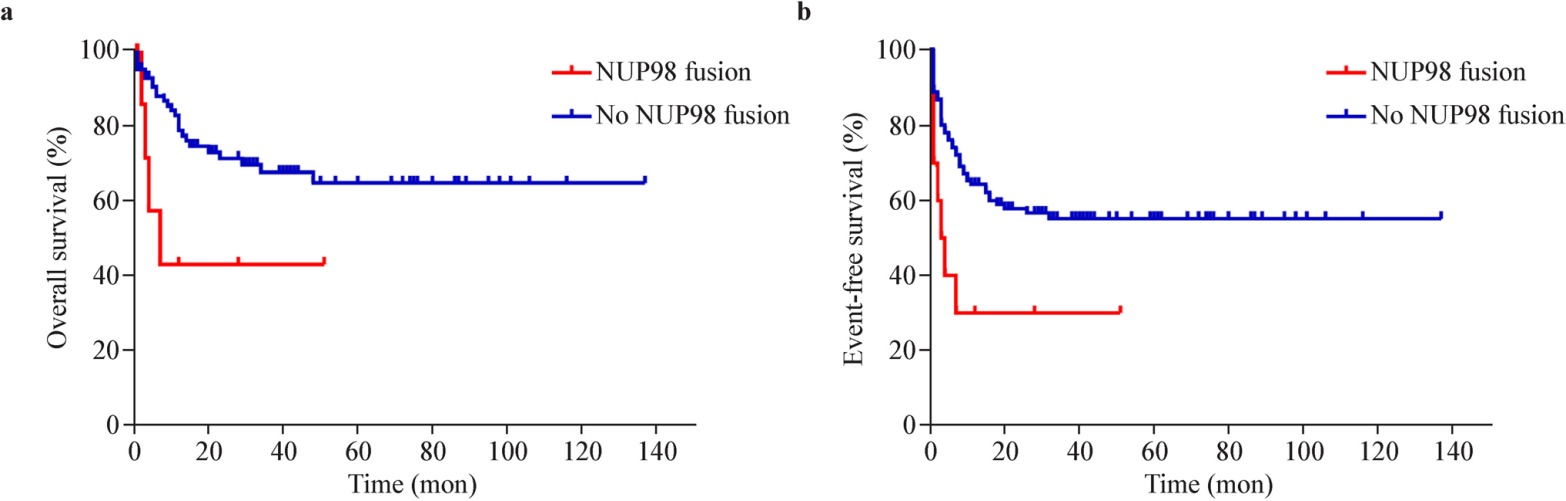
Survival of pediatric patients with NUP98-R-positive AML. **a** Kaplan–Meier estimates of OS. 3-year OS rate: 42.9% (NUP98 fusion) vs. 67.5% (No NUP98 fusion), *P* = 0.055. **b** Kaplan–Meier estimates of EFS. 3-year EFS rate: 30% (NUP98 fusion) vs. 55.3% (No NUP98 fusion), *P* < 0.05)

All 101 patients in the reference cohort received a standard chemotherapy regimen, including anthracyclines and nucleosides, during induction. Among these patients, 65 (64.3%) achieved CR after the first course (Table 1). In the NUP98-R cohort, two (20%) achieved CR after the first course, seven (70%) did not achieve remission, and one discontinued follow-up before assessment (Tables 1, 2). The CR rate after the first induction course was significantly lower in the NUP98-R group than in the reference cohort (20% vs. 64.3%, *P* < 0.05) (Table 1). The 3-year EFS rate was significantly greater in the reference cohort (55.3% vs. 30%, *P* < 0.05). Although the 3-year OS rate was greater in the reference cohort (67.5% vs. 42.9%, *P* = 0.055), this difference was not statistically significant (Fig. 2).

### Detailed case analysis

*Patient 1 (N2)*: An 11-year-old boy presented with persistent cervical lymphadenopathy for over 20 days and hematological abnormalities for two days. The laboratory results revealed a WBC count of 76.87 × 10^9^/L, a hemoglobin (Hb) level of 107 g/L, and a platelet (PLT) count of 203 × 10^9^/L. BM examination revealed 75% blasts, which were morphologically classified as M5. Immunophenotyping revealed an aberrant myeloid progenitor phenotype, with strong expression of CD33, CD123, and HLA-DR and weak expression of CD14, CD15, and CD38. Chromosomal analysis revealed a karyotype of 46–47, XY, Inc. [3]/46, XY [17]. Genetic analysis confirmed the presence of the NUP98-NSD1 fusion gene and mutations in FLT3-ITD, IDH1 and MYC. The patient was diagnosed with high-risk AML (M5).

The initial DAH regimen (daunorubicin, cytarabine, and homoharringtonine [HHT]) combined with sorafenib and venetoclax achieved CR on day 28, with an MRD of 1%. At this point, NUP98-NSD1 fusion and IDH1 mutations were still detectable, while the FLT3-ITD mutation was negative. A second course of IAH chemotherapy (idarubicin, cytarabine, and HHT) combined with sorafenib and venetoclax reduced MRD to be less than 10^–4^, NUP98-NSD1 remained positive, and FLT3-ITD and IDH1 mutations were undetectable. The patient subsequently underwent hematopoietic stem cell transplantation (HSCT) but unfortunately succumbed to a severe infection 1 month posttransplantation.

*Patient 2 (N6)*: A 5-year-old boy presented with a 1-day history of leukocytosis. Laboratory tests revealed a WBC count of 401.6 × 10^9^/L, a Hb of 57 g/L, and a PLT of 52 × 10^9^/L. BM examination revealed 93% myeloid blasts, which were morphologically classified as M1. Immunophenotyping confirmed the myeloid origin of the blasts, with strong expression of CD33, CD123, CD38, CD117, and HLA-DR. Chromosomal analysis revealed a normal karyotype (46, XY). Genetic testing revealed the NUP98-NSD1 fusion gene and mutations in FLT3-ITD and IDH1, confirming high-risk AML (M1).

The patient received induction therapy with the DAH regimen. On day 28, BM analysis revealed 8.5%, an MRD of 6.5%, and persistence of NUP98-NSD1 fusion and IDH1 mutations, while FLT3-ITD was undetectable. By day 46, disease progressed with myeloid blasts increasing to 28% and MRD also at 28%, along with continued positivity for NUP98-NSD1 and IDH1 mutations, indicating resistance to chemotherapy.

Treatment was modified to include gilteritinib, palbociclib, venetoclax, and azacitidine. By day 28 of the revised treatment, the patient achieved CR, with MRD reduced to less than 10^–4^. NUP98-NSD1 remained positive but was significantly reduced; FLT3-ITD and IDH1 mutations were undetectable. After HSCT, a 1-month follow-up confirmed CR, with an MRD less than 10^–4^ and no detectable NUP98-NSD1, FLT3-ITD, or IDH1 mutations. At the final follow-up, 243 days post-HSCT, the patient remained in CR, with no evidence of NUP98-NSD1.

*Patient 3 (N9)*: A 5-year-old girl presented with a 1-day history of leukocytosis and bilateral eyelid petechiae. Laboratory tests revealed a WBC count of 482.79 × 10^9^/L, a Hb of 73 g/L, and a PLT of 58 × 10^9^/L. BM analysis revealed 90.5% blast cells. Immunophenotyping confirmed a myeloid blast phenotype with aberrant markers; strong expression of CD33, CD34, CD123, CD13, and HLA-DR; and weak expression of CD7 and CD71. Cytogenetic analysis revealed a normal karyotype (46, XY). The patient was diagnosed with unclassified AML, with the fusion gene NUP98-NSD1 identified, and with mutations in FLT3-ITD, WT1 and CEBPA.

After DAH induction therapy, day 28 evaluations revealed BM blast cells of 35%, an MRD of 25%, and persistent FLT3-ITD, WT1, and CEBPA mutations. A second course of IAH chemotherapy reduced the percentage of BM blasts to 15% and to 3.5%, indicating a limited response. Treatment was modified to the FLAG regimen (fludarabine, cytarabine, and granulocyte colony-stimulating factor). Unfortunately, the patient died of severe infection during this treatment. Posttreatment evaluations for fusion and mutation were not performed due to financial constraints.

## Discussion

The findings of three patients with NUP98-NSD1 and FLT3-ITD mutations suggest that combining conventional chemotherapy with FLT3, BCL-2, or CDK6 inhibitors may improve patient outcomes. These patients had similar clinical characteristics, but different treatments led to different remissions and prognoses. Notably, Patient 2 and Patient 3 did not achieve remission after initial induction. Patient 2 presented a persistently high tumor burden. In contrast, Patient 1 achieved morphological remission after the first induction therapy, which included a DAH regimen with venetoclax and sorafenib. Complete morphological and immunophenotypic remission was achieved after a second cycle via an IAH regimen with venetoclax and sorafenib. This outcome suggests that combining BCL-2 and FLT3 inhibitors with the DAH regimen may have synergistic antitumor effects, which is consistent with the findings of Zheng et al. [9]. Preliminary research has indicated that the use of FLT3 or BCL-2 inhibitors as monotherapies for patients with relapsed or refractory AML with FLT3-ITD mutations may have limited efficacy. This limitation may result from tumor cell resistance to FLT3 inhibitors and the limited ability of BCL-2 inhibitors to induce apoptosis in tumor cells, which relies on alternative antiapoptotic pathways. Additionally, FLT3 inhibitors may modulate BCL-XL and MCL-1 expression, increasing the dependence of tumor cells on BCL-2 and potentially enhancing the efficacy of venetoclax [12, 13]. This mechanism may explain the greater remission observed in Patient 1 than in Patient 2.

The potential value of CDK6 inhibitors in treating pediatric AML patients with NUP98-NSD1 and FLT3-ITD mutations is significant. CDK6 is crucial for cell cycle regulation and proliferation [14]. Preclinical studies showed that CDK6 was highly overexpressed in AML tumor cells and significantly reduced after chemotherapy-induced remission [15]. Schmoellerl et al. reported that CDK6 deletion significantly reduced NUP98 fusion protein-driven leukemogenesis. NUP98-R-positive AML tumor cells also showed sensitivity to CDK6 inhibitors in both in vitro and in vivo models [16].

Tumor cells from patients with FLT3 mutations activate various signaling proteins, including STAT5, RAS/MAPK, and PI3K/AKT, along with abnormal upregulation of AURORA kinase. CDK6 inhibitors significantly reduce the mRNA levels of AKT and AURORA kinase. They achieve this by blocking the direct binding of CDK6 to these genes and modulating their transcription at promoter regions. This inhibition reduces oncogenic signaling protein expression. It elicits potent antitumor effects in FLT3-mutant AML cells [17]. Previous clinical trials have shown that CDK6 monotherapy has limited efficacy in patients with refractory or relapsed AML [14]. A common treatment regimen involves combining venetoclax with demethylating agents such as azacitidine. This combination induces apoptosis in AML cells. It activates mitochondrial pathways and reduces MCL-1 levels, thereby overcoming resistance to BCL-2 inhibitors [18]. The combination of venetoclax and azacitidine has also shown positive outcomes in elderly or frail AML patients who are intolerant to intensive chemotherapy [19]. While effective in treatment-naïve patients, its efficacy decreases in those who received prior induction chemotherapy. This may be due to reduced T-cell counts and impaired T-cell function, contributing to a poorer therapeutic response [20].

Compared with venetoclax and azacitidine, the combination of palbociclib and venetoclax had antitumor effects but did not improve outcomes [21]. Recent research has indicated that combining palbociclib, venetoclax, and azacitidine significantly enhances therapeutic outcomes in AML cells with higher CDK6 expression. Compared with venetoclax or azacitidine alone, this combination has superior antitumor effects. Palbociclib increases AML cell sensitivity to venetoclax and azacitidine by downregulating the antiapoptotic proteins MCL-1 and BCL-XL without affecting BCL-2 activity. Moreover, it also reduces the expression of other antiapoptotic proteins [22]. The three-drug regimen resulted in morphological, immunophenotypic, and molecular complete remission in an adult AML patient who relapsed with venetoclax and azacitidine treatment [23]. At our institution, Patient 2 failed to achieve remission after DAH chemotherapy, later achieved both morphological and immunophenotypic CR with a regimen that included palbociclib, venetoclax, azacitidine, and gilteritinib. Based on these studies and our case data, the combination of palbociclib, venetoclax, azacitidine, and gilteritinib showed promising potential for treating this high-risk population.

In conclusion, children with AML harboring NUP98-R and FLT3-ITD mutations often have poor responses to standard chemotherapy, leading to an unfavorable prognosis. The two treatment regimens explored in this study showed promising efficacy and may improve outcomes for this subgroup. Further validation of their efficacy and safety through multicenter clinical trials is needed. Future research should evaluate these regimens across different genetic backgrounds, focusing on achieving rapid disease remission. BM samples from these patients should be collected for single-cell sequencing to provide crucial data on pathogenic mechanisms and drug resistance. This information will aid in the development of individualized treatment strategies. Single-cell drug sensitivity analysis can help design personalized treatment regimens to achieve higher remission rates and improve long-term prognosis. These findings may enhance clinical decision-making and support the integration of precision medicine in AML treatment. Further studies are needed to validate these regimens in diverse patient populations and evaluate their long-term safety and efficacy.

## Data Availability

Access to these data can be obtained from the corresponding author upon a reasonable request.
